# A Small Acoustic Goniometer for General Purpose Research

**DOI:** 10.3390/s16050622

**Published:** 2016-04-29

**Authors:** Michael L. Pook, Sin Ming Loo

**Affiliations:** Department of Electrical and Computer Engineering, Boise State University, 1910 University Drive, Boise, ID 83725, USA; dafreakinpook@gmail.com

**Keywords:** acoustic goniometer, infrasound

## Abstract

Understanding acoustic events and monitoring their occurrence is a useful aspect of many research projects. In particular, acoustic goniometry allows researchers to determine the source of an event based solely on the sound it produces. The vast majority of acoustic goniometry research projects used custom hardware targeted to the specific application under test. Unfortunately, due to the wide range of sensing applications, a flexible general purpose hardware/firmware system does not exist for this purpose. This article focuses on the development of such a system which encourages the continued exploration of general purpose hardware/firmware and lowers barriers to research in projects requiring the use of acoustic goniometry. Simulations have been employed to verify system feasibility, and a complete hardware implementation of the acoustic goniometer has been designed and field tested. The results are reported, and suggested areas for improvement and further exploration are discussed.

## 1. Introduction

Acoustic events play an important role in communicating information about nearly any given environment. Whether the event is in a range perceivable by humans or not, many phenomena which are of interest for characterizing an environment produce a sound. A sound may hail the advent of an avalanche, the cracking of rock signaling the start of a landslide, or even the presence of gunfire. Understanding acoustic events and monitoring their occurrence can allow researchers to predict, prevent, and locate the source of interesting or potentially harmful phenomena. Consequently, research into acoustic events is a very popular aspect for many projects. Unfortunately, due to the specialized nature of many acoustic applications, no generic devices exists (aside from simple microphones or microphones tuned to specific frequencies) for conducting acoustic research. This has led to the development of many specialized sensor systems with limited flexibility. While specialized equipment serves the purpose for which it was designed, this creates a barrier to research if the scientists involved do not have the expertise needed to create their own equipment. This must be expected to a certain extent for an area of research as broad as acoustic monitoring. However, more flexible hardware designed to aid in semi-specific applications could help in furthering research.

### 1.1. Background

Processing sound and determining its cause has always played an important role in our understanding of the environment we occupy. Humans use the concept of acoustic goniometry countless times each day to solve a variety of problems. When we need another person’s attention, we call out their name, and they acknowledge us by first detecting an acoustic event and determining the source of the commotion. Excepting special circumstances, the individual can usually look directly at the source without needing to scan their field of vision. The same process can be applied in determining the source of danger, anticipating the arrival/departure of an object, and many other phenomena which produce sound. This ability which comes as second nature to us is the result of our brains automatically applying acoustic goniometry to a sensory input in order to solve a given problem.

Twenty years ago, environmental sensing research and development was reserved for corporations, governments, and universities with large budgets due to the limited availability of sensors. In recent years, commercially available sensors have become abundant for many applications. The proliferation of simple sensors has made environmental sensor system research and related fields of study more accessible to researchers with backgrounds ranging from the weekend embedded systems hobbyist to the computer engineering graduate student. This increased accessibility sparked an incredible interest in environmental sensor systems and product development which has led to a substantial growth in research. However, such growth has yet to occur for acoustic goniometer research. This useful field of study suffers from limited sensing options which require either spending a large sum or designing a custom sensor suite. A survey of the research completed shows that the majority of goniometer projects rely on very targeted, custom built sensors designed by those conducting the research (see Section 2). Very often, these systems are designed by teams with limited computer engineering experience and tend toward simpler designs which suffer from the need for large spacing between sensors (large antenna size), poor accuracy, and/or limited adaptability. Some sensor systems are commercially available but are targeted toward very specific applications and are only available to select groups at a high cost (e.g., military equipment; see Section 2).

### 1.2. Contributions

It should be noted that the smaller size pertains specifically to the field of avalanche detection in the infrasonic range. In other areas (e.g., sniper detection), smaller antenna sizes than this research have been developed. The contribution lies in the general-purpose nature, small size, ease of deployment, and low cost. Previous research in avalanche detection has shown a system with deployment of 20 m between microphones, this paper show the design of a 2 m system.

The ultimate goal of this work is to lower barriers to research in fields of study requiring the use of acoustic goniometers. Human beings in general are curious about their environment and have an insatiable desire for knowledge. When equipped with the proper tools and sufficient resources, people explore, push boundaries, innovate, and enrich the collective knowledge of the group. However, when tools are insufficient or resources are scarce, progress can be slowed significantly or even halted completely. If a researcher does not have the requisite skills or time to build their own tools, they may be forced to abandon a field of study in favor of one which is more practical for their situation. When this occurs, potentially valuable information which may have been garnered from their research may be delayed or completely lost.

In order to serve the ultimate goal of lowering barriers to research, this article focuses on the development of an inexpensive, small acoustic goniometer which is easy to deploy and capable of being adapted to meet the needs of a wide range of research applications while maintaining reasonable accuracy. Designing such a system required careful consideration of a variety of factors. The acoustic goniometer had to have the ability to work over a wide range of frequencies. Furthermore, the system needed to be simple enough for the average researcher to perform adjustments, test theories, and add capabilities. The design also had to include a structure (or example thereof) which makes deployment and use of the system as painless as possible. Each of the aforementioned design features are complex enough to consider by themselves, but each of these had to be weighed against the accuracy of the system. To achieve the best possible accuracy, the acoustic goniometer design would have had to be specifically targeted to one application with a set frequency and be implemented on the most advanced hardware locked into a single sensor configuration on a predetermined structure. However, this would have defeated the goals of this research entirely as the resulting system would have been difficult to adapt, costly, and not well suited toward experimentation and furthering research. From the other perspective, the most flexible system with the simplest user interface would not be useful in any field of research if its accuracy is unacceptably low. Thus, a balance had to be determined and practical sacrifices had to be made on both sides.

## 2. Previous Work

Since many important phenomena produce sound, the amount of research conducted in the fields of acoustic goniometry and infrasonic monitoring is not surprising. Researchers have focused on a variety of facets including applications for acoustic goniometers and infrasonic monitoring, improvements to sensor hardware, and efficient/effective firmware design [1,2,3,4,5,6,7,8,9,10,11,12,13,14,15,16,17,18,19,20,21,22,23,24,25,26,27,28,29,30]. This section details a small sample of the research that has been conducted and the technology used in the process.

### 2.1. Countersniper Research

Given the crucial need for determining sniper location (or any source of incoming fire) on battlefields, the existence of commercial products and university research created to serve such purposes is to be expected. The authors of [6] developed an acoustic wireless sensor network to serve as a countersniper system. The nodes consisted of Berkeley motes outfitted with simple sound sensors (microphones connected to amplifiers with adjustable gain) using Xilinx field programmable gate arrays (FPGAs) for high speed signal processing. The wireless network of sensors was deployed throughout a target area and communicated with a single base node. The sensors performed signal processing and handled event detection on their own. When an event (gunshot) was detected, the nodes forwarded the relevant data to the central computer for analysis. The central computer fused the data from each of the nodes that reported the event in order to calculate the location of the shooter and the direction of the shot. This design was created for the specific purpose of using acoustic goniometry to determine the location of active shooters. As such, this goniometer is not well suited to general purpose research, but it provides an excellent example of the utility of an acoustic goniometer.

### 2.2. Avalanche Detection

Van Lancker focused on the development an acoustic goniometer system to track avalanches using sound sensors in his doctoral dissertation [13]. Like [6], the hardware from his research also used a centralized computing station to handle some of the data processing. Although he experimented with several quantities of microphones, each of his sensor nodes used four microphones in an “Echo Star” pattern with a spacing between 20–35 m. He successfully designed, built, and deployed his sensor networks for several applications including a 4 year study of avalanche detection/monitoring in Switzerland. His research spawned what is known as the ARFANG Station in Switzerland which currently monitors avalanches in the Alps. Van Lancker’s work was one of the biggest boons to research in this field. His design laid the ground work for better understanding the design and implementation of such systems. Since the publication of his work, newer technologies have created the potential to make smaller, less expensive designs possible. At the time of his research, microprocessors did not have the ability to sample and process data fast enough to allow for small inter-sensor spacing. Thus, the prototype needed to employ a large, expensive computer to accomplish the processing tasks within the requisite amount of time. Additionally, due to sampling frequency restraints, Van Lancker’s work focused on increasing inter-sensor spacing as much as possible to improve precision which essentially sacrificed ease of deployment for the sake of functionality. The improvements in modern microprocessors have rendered such decisions unnecessary. With ADC sample rates currently reaching into the megahertz range for inexpensive processors and well above that for FPGAs, the only limitation current hardware places on goniometry development is the speed at which the data can be processed and stored. Thus, taking advantage of modern technology can improve the ease with which researchers deploy acoustic goniometers while maintaining the same level of accuracy as previous systems. One goal of the current research was to prove the validity of this statement by developing such a system.

## 3. Acoustic Goniometer Theory

Since goniometry is derived from the combinations of two Greek words meaning “angle” and “measure,” the definition of goniometer as “a device which measures angles” is fairly obvious. An acoustic goniometer, therefore, is a device which measures angles using sound. Such a device usually includes two or more sensors capable of detecting sound spaced apart at a known distance. Each sensor is equipped with a synchronized clock allowing the goniometer to detect the differences in the time at which a sound event is detected by each sensor. This information is used in conjunction with the sensor spacing and the constant speed of sound to determine the differences in distance between the sound event source and each sensor. With the aid of basic trigonometry, this result can be used to determine the direction of arrival (DOA) for the sound event. As mentioned in Section 1, this process of acoustic goniometry is used regularly by the average person and is usually taken for granted. However, similarly to other abilities with which humans (and many animals) are innately endowed, the process is challenging to implement in an embedded or even computer algorithm. This section explains the process used to perform acoustic goniometry in detail. The derivations and explanations found in this section are not an original work but were gleaned mainly from [13,31].

**Figure 1:**
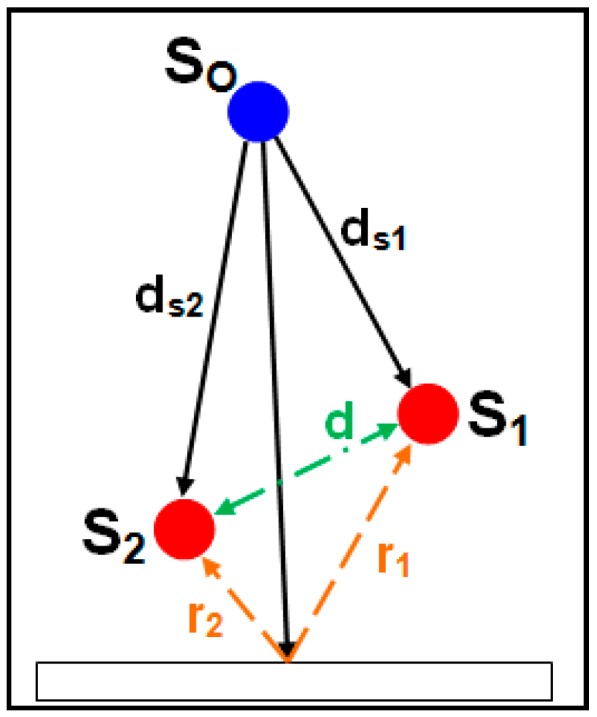
Acoustic goniometer sensor pair diagram.

Consider the simple acoustic sensor system as shown in Figure 1 where the two sensors (S_1_ and S_2_) are represented by red dots spaced apart by some distance (d). Now, suppose an event occurs producing sound at the location indicated by the blue dot (S_O_). For ease of explanation, assume the path between the sensors and the sound source is unobstructed. Further, given a large enough distance between the source and the sensors (represented by d_s1_ and d_s2_), note that even a sound source with multiple related origins (e.g., an avalanche) can be modeled as a perfect point source whose sound radiates outward equally in all directions. The only remaining difficulty with this model is the presence of reflected signals (see r_1_ and r_2_ in Figure 1). Reflections occur when a sound event reverberates off an object along its path and can make the process of event detection more difficult by masking the fingerprint of a given event. For the sake of explaining goniometry by itself, assume reflections are not present in the current model (exclude r_1_ and r_2_).

Under the conditions given above, the time required to travel from S_O_ to S_1_ can easily be calculated by the casual observer using Equation (1) where c is the constant speed of sound. The time required for the sound to travel from the source to the second sensor can be defined in a similar fashion. However, since the goniometer is trying to calculate the direction from which the sound event originated, it obviously has no knowledge of d_s1_ or d_s2_. Equation (1) can be modified instead to give the relationship between the difference in the distances between the sensors and the source (Δd_12_) and difference in propagation times (Δt_12_) from the source to the sensors (as shown in Equation (2)).

(1) $$t_{1}=\frac{d_{s1}}{c}$$

where t_1_ is the time taken for the sound wave to reach S_1_, d_s1_ is the distance from the source to S_1_, and c is the speed of sound:

(2) $$\Delta t_{12}=t_{2}-t_{1}=\frac{d_{s2}-d_{s1}}{c}=\frac{\Delta d_{12}}{c}$$

where t_2_ is the time taken for the sound wave to reach S_2_, and d_s2_ is the distance from the source to S_2_.

**Figure 2:**
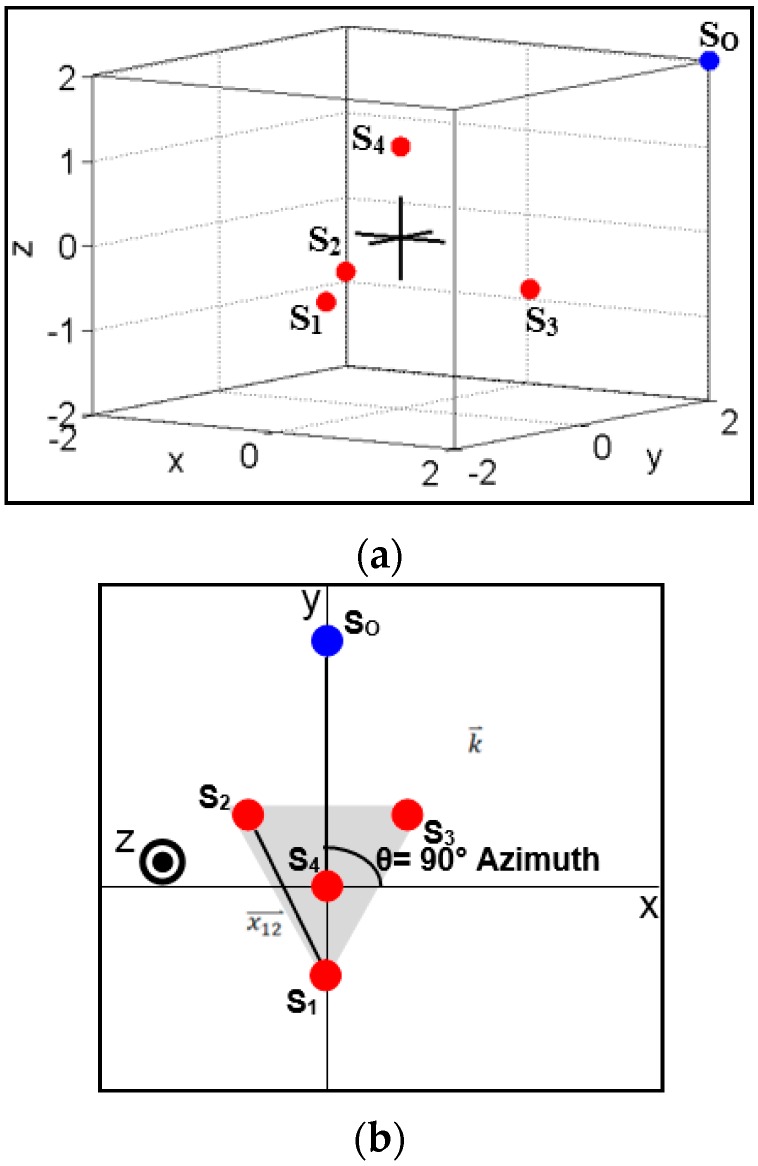
Acoustic goniometer vector diagram**;** (**a**) Sensor orientation 3D view (origin marked with 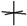); (**b**) Top-down view with vectors.

In order to continue with the derivation, a more complex goniometer model with a coordinate system must be considered (see Figure 2). The new model shows a goniometer with four sensors where S_4_ is located along the positive z-axis, and the other three sensors are located in a plane parallel to the one defined by the x and y coordinate axes in the negative z-axis. The sensors are arranged such that the center of the goniometer is located at the origin. The wave vector ($\vec{k}$) is defined as the sound event’s path of travel from the source to the center of the goniometer. Thus, the vector defining the path from S_1_ to S_2_ ($\vec{x_{12}}$) is related to the normalized wave vector ($\vec{n}$: Equation (3)) by Equation (4). Although the time required to travel from the source to the respective sensors (t_1_ and t_2_) is not known by the goniometer, this difference can be obtained by simply using the difference between each sensor’s timestamp of the sound event:

(3) $$\vec{n}=\frac{\vec{k}}{\left|\vec{k}\right|}$$

where $\vec{k}$ is the sound wave’s path of travel from the source to the center of the goniometer, and $\vec{n}$ is the normalized vector:

(4) $$\Delta t_{12}=\frac{\vec{n}\cdot \left(\vec{x_{2}}-\vec{x_{1}}\right)}{c}=\frac{\vec{n}\cdot \vec{x_{12}}}{c}$$

where Δt_12_ is the difference in arrival times at the sensors, x_1_ and x_2_ represent the positions of S_1_ and S_2_ (respectively), and c is the speed of sound.

Now, if Equation (4) is used for each of the sensor pairs, a system of equations may be defined to solve for $\vec{n}$ (see Equation (5)). In addition to giving the distances between sensors pairs, the D matrix also determines the coordinate system used by the acoustic goniometer. Since D is a matrix of vectors rather than magnitudes, the direction (polarity) of the vectors determine the coordinate axes. In order to solve Equation (5) for $\vec{n}$, the inverse of the matrix D must be calculated. This can be done simply if D is invertible. However, this only occurs if three sensor pairs are used (assuming the calculations are taking place in 3-D space). Otherwise, a pseudo-inverse of D must be calculated using Singular Value Decomposition (SVD). Equation (6) shows the inverse calculation and the final solution to solving Equation (5) for $\vec{n}$. In Equation (6), D_p_ represents the pseudo-inverse of D:

(5) $$T=\;\frac{\vec{n}\cdot D}{c}$$

where D is a matrix containing the vectors between sensor pairs, and T is a matrix containing the time delays between sensor pairs:

(6) $$\vec{n}=c\cdot {\left(D^{t}\cdot D\right)}^{-1}\cdot D^{t}\cdot T=c\cdot D_{p}\cdot T$$

where D^t^ is the transpose of D, and D_p_ is the pseudo-inverse of D.

**Figure 3:**
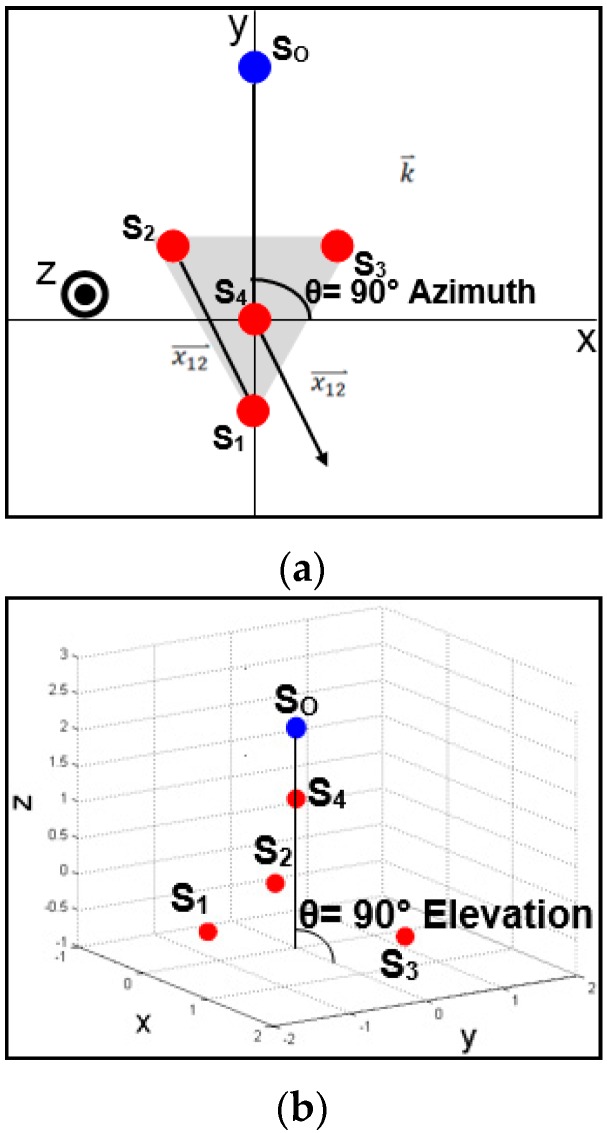
Example source locations explaining azimuth and elevation; (**a**) Source location at 90° azimuth 0° elevation; (**b**) Source location at 0° azimuth 90° elevation.

Once the normalized wave vector is calculated, the DOA azimuth (A) and elevation (E) can be determined using Equations (7) and (8), respectively. The equation for azimuth provides the angle with respect to the the x-axis of the goniometer’s coordinate system. Thus, a sound wave traveling along the y-axis would correspond to an azimuth of 90° (see Figure 3a). The elevation equation provides an angle with respect to the y-axis of the goniometer’s coordinate system. Therefore, a sound wave traveling directly down the z-axis would correspond to an elevation of 90° (see Figure 3b). The choice for these conventions can change depending on the needs of a given project and can be adjusted easily with minor modifications to Equations (7) and (8):

(7) $$A=90{}^{\circ}-\tan^{-1}\frac{n_{x}}{n_{y}}$$

where A is azimuth, and n_x_/n_y_ are the first and second elements of the normalized wave vector:

(8) $$E=-\tan^{-1}\frac{n_{z}}{\sqrt{n_{x}^{2}+n_{y}^{2}}}$$

where E is elevation, and n_z_ is the third element of the normalized wave vector.

The importance of antenna geometry to acoustic goniometry is made fairly clear in the work of Van Lanker. In his dissertation, Van Lanker explored the use of various geometries and made recommendations for particular geometries depending on the type of phenomena and associated frequency being monitored [13]. While the goal of the current work was not to find the best geometry for any particular application, providing some information on constraints of selecting a geometry is relevant to a theory of acoustic goniometer operation. A simple analysis of Equations (6)–(8) quickly yields the most important constraint on selecting an antenna geometry. A goniometer attempting to locate the source of a sound occurring in 3-D space must have an antenna geometry which occupies 3-D space. In other words, an antenna which has no sensors whose positions vary in the vertical axis will not be capable of calculating the elevation for a DOA vector. Similarly, a geometry which resides completely in a plane of either the x-axis or y-axis would be unable to calculate the azimuth for a DOA vector. To understand this, consider Equation (8). If the z-axis term of the normalized vector $\vec{n}$ is zero, the calculated elevation will likewise be zero. One way this is guaranteed to always occur is for the differences in z-axis distances between the sensor pairs to be zeros (all sensors in the same z-plane). Thus, if all of the sensors of the goniometer antenna are aligned in the z-axis, the calculated elevation from the goniometer will always be zero. A similar analysis holds true with regard to the calculated azimuth for Equation (7) and an antenna whose sensors are all aligned in either a common x-plane or y-plane.

## 4. Implementation

**Figure 4:**
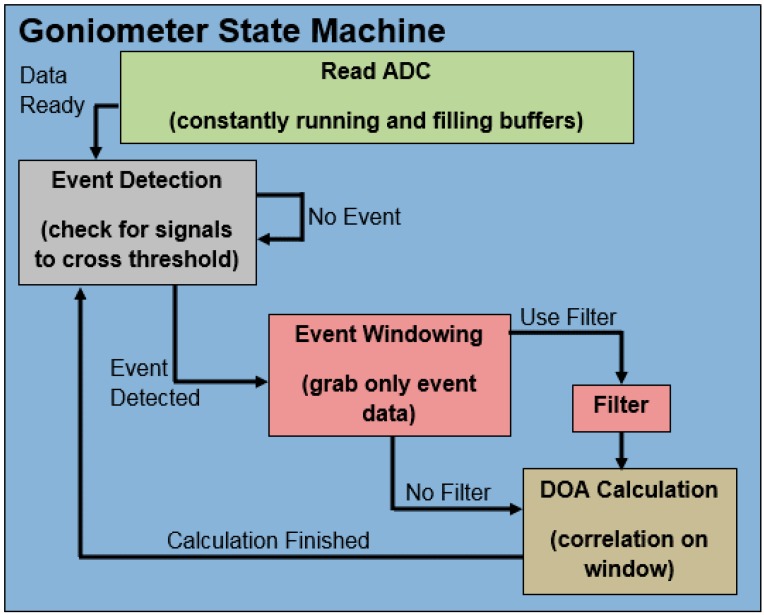
Goniometer state machine.

For the sake of discussing the design of the system, the acoustic goniometer can be split into two main components: firmware and hardware. Each of these can be further subdivided into smaller components/modules which carry out the task of collecting data, detecting events, and determining the location of event sources (DOA calculation). Figure 4 shows a basic block diagram of the acoustic goniometer components. The hardware acts as the conduit for the firmware to the outside world for both collecting information and providing feedback/data. Hardware includes the platform (processor and associated circuitry), the sensors, and the mechanical systems. The firmware collects, analyzes, and stores data provided by the hardware. To accomplish this, individual algorithm modules work in concert to schedule system tasks (operating system), manage/store incoming data (ADC Reader), and provide/record data analysis (event detection, event windowing, and goniometer state machine).

### 4.1. Firmware

The design of the acoustic goniometer firmware was modeled in the simulation code. However, the two implementations differ in one very important way: The simulations ran on a computer analyzing static data stored in an array, while the hardware has to be capable of performing in real-time. As such, the simulations could be broken down into individual functions called at will by the researcher and stopped for debugging or adjustment at any point during the course of the simulation. The embedded implementation, however, had to be designed to run without interruption, switching automatically between tasks as events are detected all within a limited amount of time due to the real-time processing requirements demanded for the sensing task. Thus, although the simulations were able to prove feasibility and test potential algorithms, actual implementation of the goniometer on embedded hardware required careful development of low-level hardware drivers, support devices, scheduling, and memory management.

#### 4.1.1. Goniometer State Machine

As mentioned earlier in this section, the main source of challenge associated with implementing the acoustic goniometer in hardware as compared to Matlab simulations is the real-time nature of the embedded implementation. The embedded system can’t wait for input from the researcher to validate its event detection or correlation results and must decide on its own when to move between steps in the calculation. Each step in the goniometer’s process must wait to process data until it has sufficient/valid data. In turn, each step must either trigger the next in line or restart the process to detect and process future events. The process of determining the direction of arrival for a sound event on the embedded platform has been split into three steps (or states): Event detection, event windowing, and DOA calculation. Figure 4 shows the states for the goniometer state machine. Once an event is detected, the second state stores a window of the data from each sensor. This window of data is then used by the third state to calculate the DOA for the given sound event.

##### Event Detection

Every decision made during the design and implementation of an embedded system is a balance of tradeoffs, and the event detection stage is a shining example of this rule. This portion of the goniometer state machine had a considerable number of implementation options each with their own set of advantages and disadvantages. The simplest algorithm to implement is a threshold event detection scheme. The advantages of this design are its low cost in terms of processing time and the ease of implementation. However, this solution suffers from the inability to distinguish between complex events. Unless amplitude is the only feature which marks the difference between signals of interest, the threshold design can’t be used to determine what caused the sound event. Unfortunately, this is rarely the case. The second method considered, fingerprinting, can be used to differentiate between signals of interest. However, depending on the method employed, the math used to search through raw data for a particular fingerprint can become quite complex and require an untenable number of processor clock cycles. As an example, consider the use of relative minima and maxima (peaks) for fingerprinting events of interest. The algorithm could employ a combination of amplitudes and the number of peaks to differentiate between events. A technique this simple could be implemented in hardware without a problem in the current system. However, a more complex fingerprinting technique operating in the frequency domain or performing real-time correlation on all raw data would not be a viable option. Since, the simple fingerprinting algorithm would only be effective if such features were the defining difference between events of interest, an algorithm whose complexity fell between these two extremes might need to be designed if a fingerprinting method was deemed necessary. The defining features which separate events of interest can change with each application/environment. Thus, specific applications for the acoustic goniometer must use the option which provides acceptable accuracy while minimizing the strain on the processor. Many options exist for creating such algorithms and should be explored for the sake of improving system performance and flexibility.

The final decision for testing the goniometer hardware was made with expediency and the capabilities of the hardware as the primary factors of concern. The event detection state is the only part of the goniometer state machine which processes all incoming data. As such, performing complex math operations as part of the event detection state was deemed inadvisable. Changing the processor to a more capable piece of hardware or adjusting the event detection state to subsample incoming data could allow for more complex algorithms to be employed. The current algorithm employs a simple comparison to a threshold value stored on the SD card. Every measurement from the sensors is checked against the threshold set by the researcher. Once one of the sensors output values falls below the threshold, a flag is set which moves the system to the next state.

##### Event Windowing

**Figure 5:**
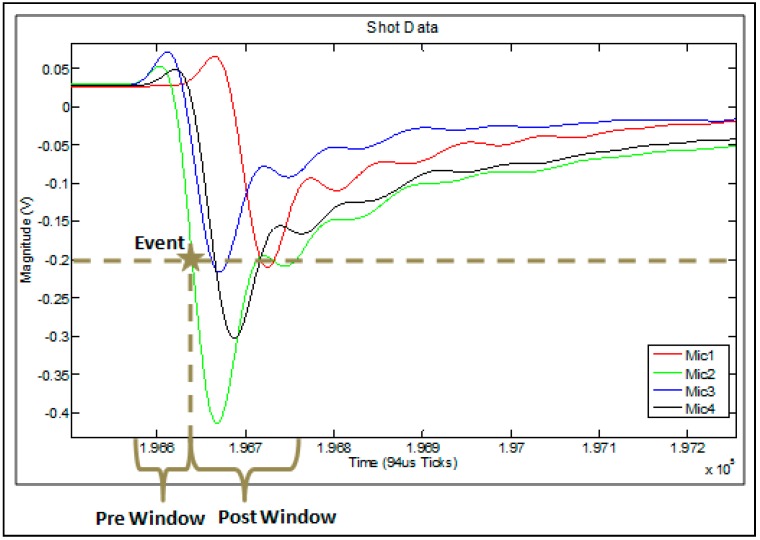
Event window parts.

An event window in the current algorithms is split into three distinct parts as shown in Figure 5: Pre Window, Event, and Post Window. The event shown in the figure is registered when one of the sensor signals crosses the threshold (shown as the Mic2 signal crossing the dotted line). While crossing the threshold defines the event, this does not indicate the start of the event. In order for the correlation algorithm to be accurate, the start of the event must be included in the window. Thus, a section of the data prior to the event (designated as Pre Window) makes up the first part of the event. The event itself (crossing the threshold) is the second part. Finally, notable features of the event must also be included in order for the correlation algorithm to work, so a “Post Window” of sufficient length to include at least the first minimum is included in the event window as well.

The event windowing state is the most lengthy in the goniometer state machine. Unlike the simulations, the firmware implementation of this algorithm is handicapped by the real-time data collection process. The simulations were made simpler by the fact that all of the data had been collected in advance. Further, all of the simulation data was held in a single multi-dimensional array. For the actual hardware, data had to be processed as it was collected in multiple single dimension arrays shared by all four sensors. Splitting the data across multiple arrays created several windowing scenarios which had to be handled by the embedded system. The best case scenario is one in which the sound event occurs somewhere near the middle of one buffer allowing for a sufficient number of samples before and after the event occurred to provide data for the full length of the event window. In this situation all of the data needed for analysis can be analyzed with minimal indexing complications. However, given the size of the event windows needed for the test scenarios and the limited length of the sensor buffers, this case is somewhat rare. A far more likely occurrence is the situation where data must be taken from two sample buffers in order to obtain a full window of the event. If the sound event occurs near the beginning of a sample buffer, data is needed from the end of the previous buffer to fill the event Pre Window. However, if the sound event occurs near the end of a sample buffer, data must be collected from the next sample buffer in order to fill the Post Window. Getting data from the next buffer has an added complication that the next buffer may not be ready. In this situation, the state machine must save its place and release the processor until data is available.

Taking so many possibilities into account requires careful handling of indexes and a significant amount of processor time. Data selected for a window must be copied from the sample buffers in order to free the buffers for storing the next samples. Additionally, all digital filtering must be completed during the windowing stage to keep the strain on system resources to a minimum. One could argue that, since each sample must be compared against the event threshold, filtering during the event detection stage makes the most sense. However, doing so would dramatically increase the number of computations as this would apply the filter to all of the data collected by the goniometer. By performing the filtering in the windowing state, filtering is only applied to data of interest, and the vast majority of the unused collected data is left unfiltered (reducing processor strain).

##### DOA Calculation

The DOA calculation portion of the state machine handles all calculations specific to the acoustic goniometer. While event detection and windowing could be applied to most event sensing applications, the correlation and angle calculations performed in this state are specific to goniometry. This part of the state machine is split into three sections. The first of which simply calculates the averages (DC offset) of the sensor data in order to subtract it from each of the signals. Once the averages are determined, the second part of this state performs a correlation between the sensor pairs to determine the difference in arrival times at each sensor. Finally, the third portion of the DOA state uses the equations described in Section 3 to calculate the azimuth and elevation for the sound event.

### 4.2. Hardware

The hardware used to prove the feasibility of the acoustic goniometer can be split into three basic parts: The platform, the sensor, and the antenna. The platform consists of the main processing unit and circuit board. A sensor includes the microphone and associated circuitry used to sense acoustic events. Finally, the antenna is the system as a whole including geometric layout and support structure. The next three sections discuss each portion of the hardware as well as any potential improvements.

#### 4.2.1. Platform

**Figure 6:**
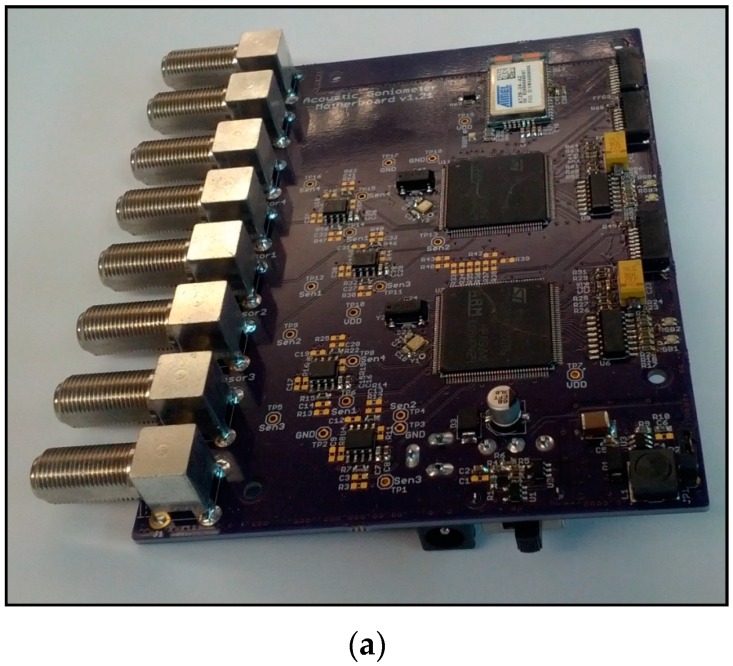

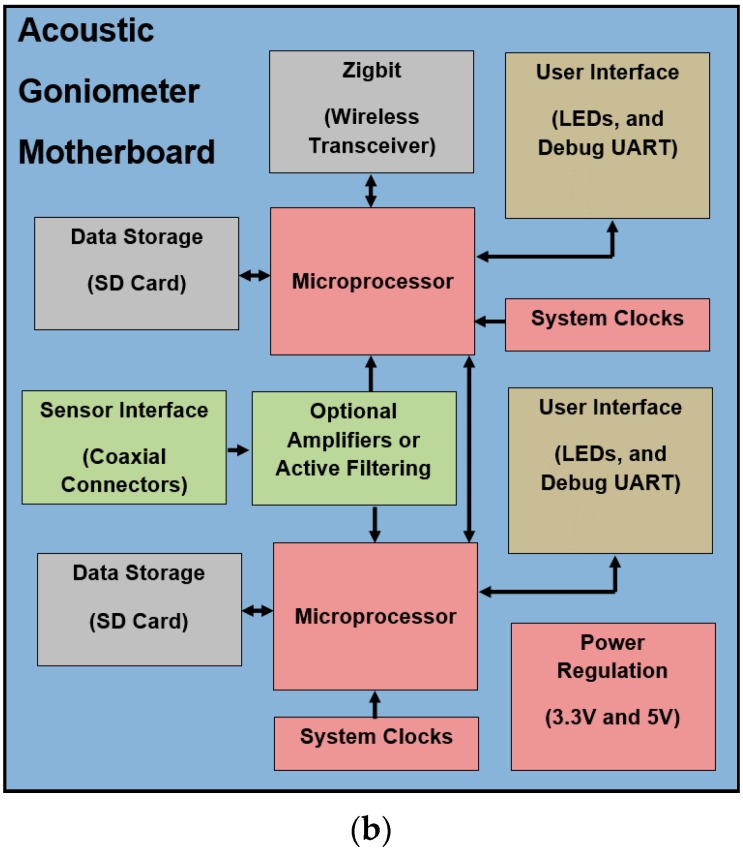
Acoustic goniometer motherboard; (**a**) final acoustic goniometer motherboard; (**b**) motherboard block diagram.

A complete implementation of the acoustic goniometer has been realized (see Figure 6). The ARM M4 CPU used in this hardware is more than capable of handling the goniometer calculations for most low frequency applications. However, due to timing constraints and the desire to record the raw data, the final design includes 2 processors: one to handle goniometry calculations and one to handle data storage. Writing data to a storage medium is a lengthy process (by microprocessor standards) even for small amounts of data and is even more cumbersome for the vast quantity of data recorded by the goniometer. While the processor is busy communicating with the storage medium, little else can be accomplished. Consequently, since the goniometer calculations require a significant amount of processor resources, one microprocessor can’t handle both tasks efficiently unless a more capable/expensive processor is selected. Mitigating the impact of data storage in order to implement the system on a single processor is possible but not advisable at this stage of the research due to the sacrifices which must be made to bring such an implementation to fruition. Instead of recording all raw data, the goniometer could subsample the raw data thus storing only a small portion. In theory, a goniometer interested in the infrasound range could easily sample the data at rates as low as 200 Hz and still be able to reproduce the signals of interest. This would reduce the number of stored samples by a factor of at least 50. Such significant savings could allow the system to operate with a single processor. However, such an algorithm would significantly increase the amount of error present in the DOA calculations. While the signals could be reproduced (in theory), the key requirement of operation for the DOA calculations is the determination of the difference in TOA at each sensor. This ability is predicated on the ability of the system to accurately determine the TOA for an event. As the sample rate decreases, the acoustic goniometer’s TOA determination becomes less accurate and the overall performance of the system suffers. Additionally, in order to validate the simulations and verify the functionality of the hardware, the raw data from the sensors must be collected and analyzed. Once the goniometer calculations have been proven satisfactorily accurate, this algorithm change (or similar ones) could potentially be used to reduce the system to one microprocessor as long as the lowered system accuracy was acceptable for the given application. As another option, the recording algorithm could be made to store data only for events further reducing the number of required writing transactions with the storage medium. This could mitigate the cost of storing data sufficiently to allow a single processor to handle the full goniometer implementation without negatively impacting the performance of the system. The sacrifices made to realize this system should, however, be noted.

While the dual processors do increase the cost of the system, the added utility and expandability they provide should not be discounted. Although the previous discussion suggests ways in which the system could operate on a single processor, the intent was not to preclude researchers from exploring the idea that a dual processor system enables the possibility of cooperative processing. The hardware has been designed such that three communication protocol signals connect the two processors (UART, I^2^C, and SPI). This feature was added to allow for data sharing between the two processors with the idea that cooperative parallel computing techniques might be a beneficial avenue to explore.

In addition to the twin processors, the current Acoustic Goniometer Motherboard design includes two other features of interest. The first is the reconfigurable voltage followers that connect the sensor signals to the processor ADCs. These were designed to allow for active filtering or provide additional gain as demanded by a given application. In the default configuration, they merely provide a high impedance buffer to prevent the sampling process from affecting the signals. The second feature of interest is the inclusion of a Zigbit radio. The radio uses the Zigbee communication protocol allowing it to form wireless mesh networks. This feature makes the system capable of being used as part of a wireless sensor network for more ambitious, large-scale projects. Wireless communication could provide a way to confirm events, pinpoint locations of events, and apply data fusion techniques with other sensors to gain more complete environmental data.

#### 4.2.2. Sensor

Due to the wide array of applications requiring microphones and the availability of many publications on sound sensor design (e.g., [13]), creating an acoustic sensor for a goniometer can be a fairly simple task. The primary consideration is the target frequency range as determined by the acoustic signature of the desired event. Once the range of interest is known, a microphone may be selected with an appropriate frequency response, and any filtering and amplification circuits can be designed. The only potential complications which arise in the course of this process are caused by limitations imposed by the system specifications (e.g., cost, sensor adaptability/flexibility). Since the current research goals required the goniometer to adapt to a wider range of applications while maintaining as low of a cost as possible, the sensor design process included a few extra challenges.

The most difficult and important part of designing a flexible acoustic sensor was the selection of the microphone. Given that the frequency range of interest for the bulk of this work was in the infrasound range, the option of picking a specialized infrasound microphone was tempting. However, taking this shortcut would have significantly increased cost since these microphones are typically sold for $1,000–$2,500 each (as of the time this paper was written). Aside from this issue, picking specialized microphones could easily have led to an implementation dependent on specialized microphones for each application. Thus, the decision was made to use a simple electret condenser microphone with a wide frequency range. Although inexpensive electret microphones do not typically have a flat frequency response in the infrasound range, other researchers indicated that such devices could be used with reasonably accurate results in infrasonic sensing applications [13]. Since a specific frequency range of interest had been defined for the current research, the only other drawback to choosing the inexpensive microphone option was its response to a wide range of frequencies. However, this problem is easily solved using analog filtering, digital filtering, or a combination of the two. Since filtering is usually desired for any sensing application (even with specialized microphones), the inclusion of filters does not preclude the use of any microphone and only serves to improve sensor flexibility.

**Figure 7:**
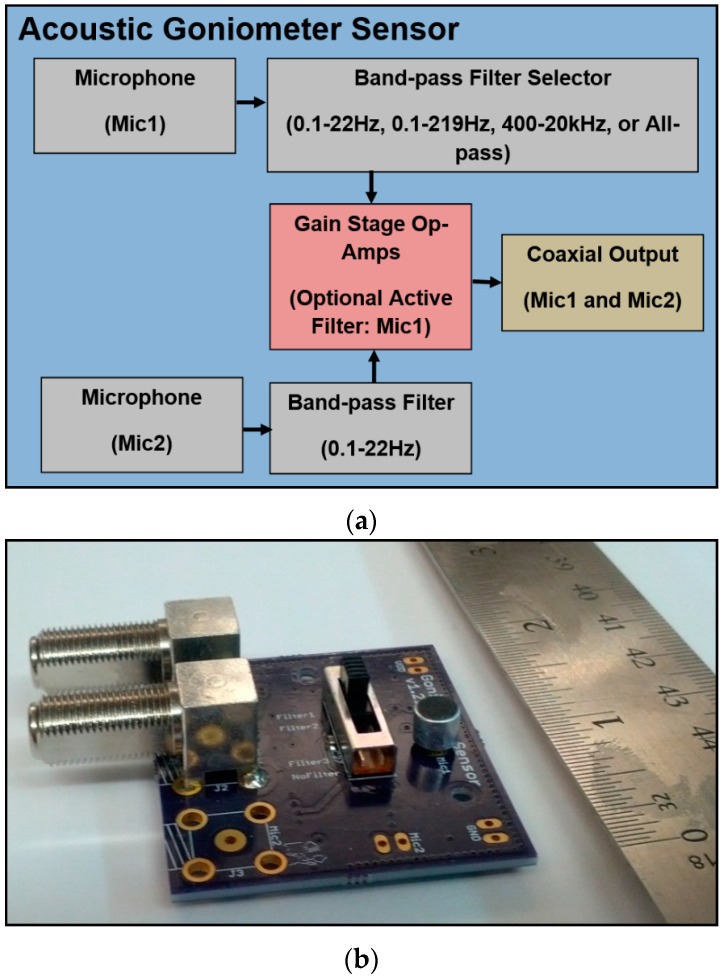
Final prototype acoustic goniometer sensor; (**a**) Final sensor block diagram; (**b**) Final sensor board (~2 × 2 inches).

While the initial prototype served its purpose admirably and helped prove the feasibility of the design, the sensor needed several improvements before the implementation could be considered finalized (see Figure 7). One such point of improvement to increase the sensor’s flexibility was the filtering system. As mentioned previously, the initial prototype acoustic goniometer used fixed hardware filters to remove undesirable noise and select the frequency range of the microphone. These filters could be adjusted by changing resistor values and configurations given a little effort and a certain amount of soldering ability. However, such adjustments would be difficult for a researcher not versed in electrical engineering concepts and could be made easier by making adjustments to the initial design. The possibility of removing all analog filters in favor of completely digital implementations was considered. This option appears attractive since it allows for the most flexibility. However, doing so would mean moving the filtering firmware from its current location in the goniometer state machine (after event detection) to the raw data processing stage in order to provide the frequency selectivity currently handled by the analog filters. As such, the processor would have to perform the filtering operation on all raw data dramatically increasing the number of required floating point operations and negatively impacting the responsiveness and performance of the goniometer. Thus, the microphone hardware was modified to include an analog filtering system with selectable frequency ranges. In order to make the system more customizable for varying research needs, the method of filter selection chosen was a simple slide switch. The final design is shown in Figure 7. The design includes a double pole four throw switch which selects the band-pass filter for the “MIC1” signal. The two poles of the switch connected to the microphone and the input of the operation amplifier can be connected to the inputs and outputs of four analog filters with pre-determined ranges. The purpose of the analog filter is not to provide a perfectly clean signal but only to limit the frequency range of the microphone. Thus, pre-determined ranges can be safely used without damaging the flexibility of the system. The default configuration of the sensor includes 3 band-pass filters and 1 all-pass filter. The ranges on the band-pass filters are set for low frequency infrasound (0.1 Hz to 22 Hz), wide range infrasound (0.1 Hz to 219 Hz), and audible frequency (400 Hz to 20 kHz). A simplified explanation of the final sensor design can be seen in Figure 7a.

#### 4.2.3. Mechanical Systems

While the bulk of this research is focused on the hardware and firmware development, the design of an acoustic goniometer requires a significant amount of mechanical design as well. In order to test the hardware and firmware, a sensor configuration including the number of sensors, the geometry of the antenna, and inter-sensor spacing had to be selected. Additionally, a practical structure had to be created for sensor deployment. Enclosures also had to be created to protect against environmental hazards damaging the goniometer as well as to prevent interference with data acquisition and analysis.

##### Antenna Design

Designing an antenna for an acoustic goniometer is a challenging problem dependent largely upon the application of interest. The key aspects of an antenna design which must be addressed are the number of sensors, the geometry of the antenna, and the spacing between each sensor. Other points of concern are minor and are usually seen with any deployed sensor system (e.g., case design, material used for structure, method of mounting, *etc.*).

Determining the number of sensors required for an acoustic goniometer is a fairly straight forward task. If the device is to be used in a study only concerned with two dimensional space (no elevation), the minimum number of sensors is three. However, if elevation is of interest (as with the current research), at least 4 sensors are required. These rules supply a minimum number, but more sensors may be added to improve the reliability of the data or the accuracy of the calculations. Additional sensors may be used to provide alternative sensor pairs if correlation between some sensors does not provide trustworthy results (see example in Section 4.2: The analysis of Figure 7). According to Van Lancker, depending on the processing method, an increased number of sensors can be used to improve the signal to noise ratio of the cross correlation calculation [13]. However, an increase in the number of sensors makes antenna deployment more challenging, increases the cost of the goniometer, and comes at a cost in processing time due to the increased number of floating point calculations required to include more sensor pairs. Thus, the goal of any antenna design should be to use the minimum number of sensors required to maintain the desired system performance.

In his master’s thesis, Van Lancker researched antenna geometry for acoustic goniometers extensively [13]. According to his work, antenna geometry for a given goniometry application is determined by the characteristics of the source to sensor configuration (*i.e.*, the expected angles at which the sound events will be received by the antenna). For example, the most important factor concerning the design of the acoustic sensors which affects the design of the antenna is microphone directionality. If the microphones are directional, the antenna geometry must be selected with this in mind. When an expected direction of arrival is known for an application, directional microphones can be used to increase the performance of an antenna geometry which favors the angles of interest. However, if such is not the case, the antenna geometry must make up for the directionality of the microphones in order to avoid favoring particular DOA calculations with greater accuracy than others.

As with the other two aspects of antenna design, the research performed by Van Lancker provided useful insight into the selection of inter-sensor spacing. Assuming the limited resolution of an actual hardware implementation is not imposed (*i.e.*, infinite precision sampling and calculations are used), the initial assessment may be made that larger inter-sensor spacing should always produce more accurate results since larger distances between sensors produces greater differences in arrival times at sensors improving the effectiveness of correlation calculations. However, this is only the case to a certain extent. In order to satisfy Nyquist and avoid anomalous data, the sensor spacing must be less than half the wavelength of the acoustic event of interest [13].

##### Structural Design

**Figure 8:**
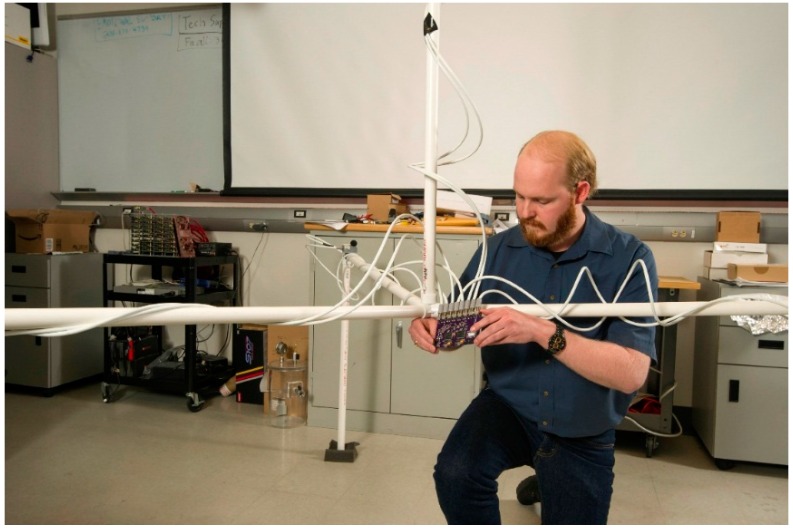
Acoustic goniometer antenna prototype.

Since the purpose of the prototype was to prove the feasibility of the system in general, the antenna geometry was selected with ease of implementation in mind. The antenna design used by the current research was modeled after the geometry explained by Figure 5. Four sensors were attached to a PVC frame in a regular tetrahedral pattern with an approximate spacing of 2 m. The fully assembled antenna can be seen in Figure 8. The PVC frame was designed to make assembly and disassembly as simple as possible in order to allow for rapid deployment and testing of the prototype. In designing for ease of deployment, certain sacrifices were made in the realm of durability and system performance. In the current setup, the sensors are held in place by tape at measured intervals along the PVC frame. The measurements must be done at each deployment and are not exact. Further, give in the tape allows for small movements in the sensors after they are placed. Another problem with the spacing can be seen clearly in Figure 8. Notice that sensor 4 at the apex of the tetrahedron is taped to the PVC on the side closest to the camera. This places the sensor closer to sensor 2 (see sensor on the right side of the figure) than sensor 1 (rear sensor in the figure). Although each of the mentioned problems with sensor spacing is minor, they still introduce a source of error that could be removed with a better antenna design. As for the durability of the antenna, PVC is fairly flexible and the joints were not sealed. Consequently, the structure was able to sway and bend in the wind going so far as to move in significant gusts. While this was acceptable for the testing phase, a more solid material than PVC with more exact sensor spacing should be created for the final design. A more rigid material should be selected, and the antenna should be anchored to the ground with either stakes (for a less permanent deployment) or concrete (for a more permanent solution).

##### Enclosure Design

**Figure 9:**
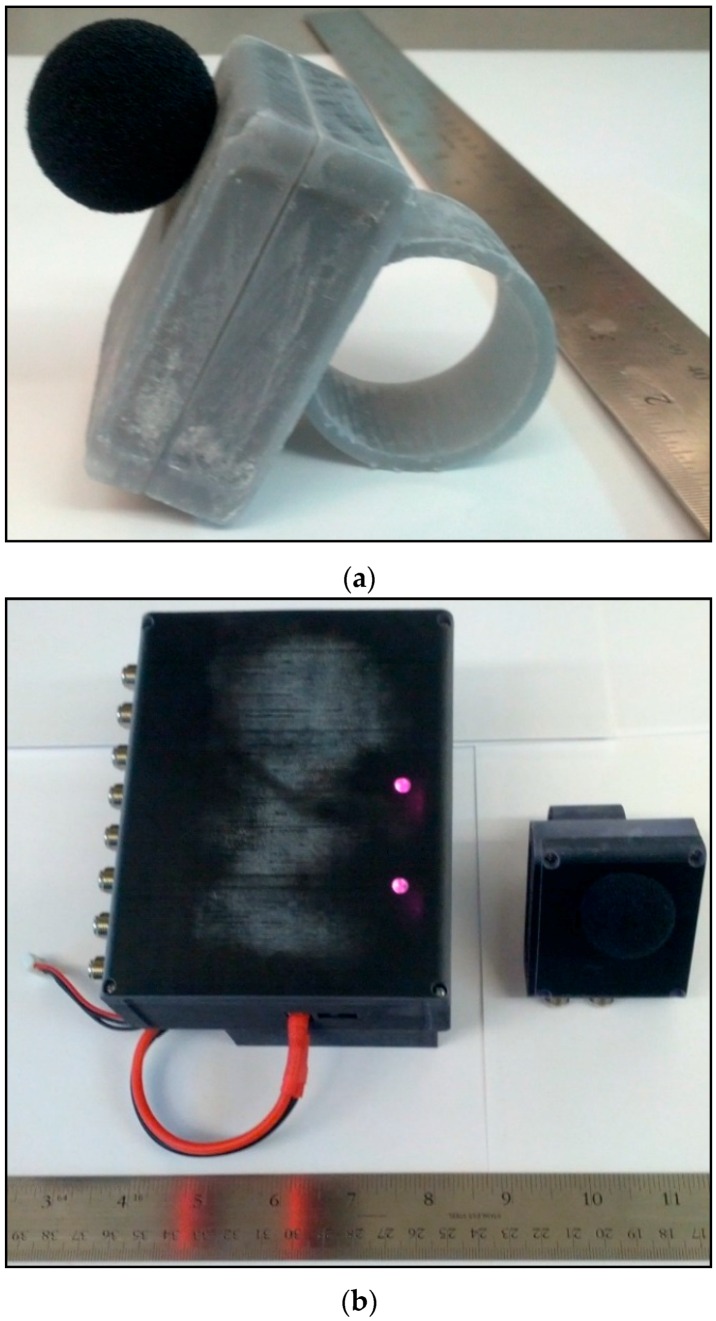

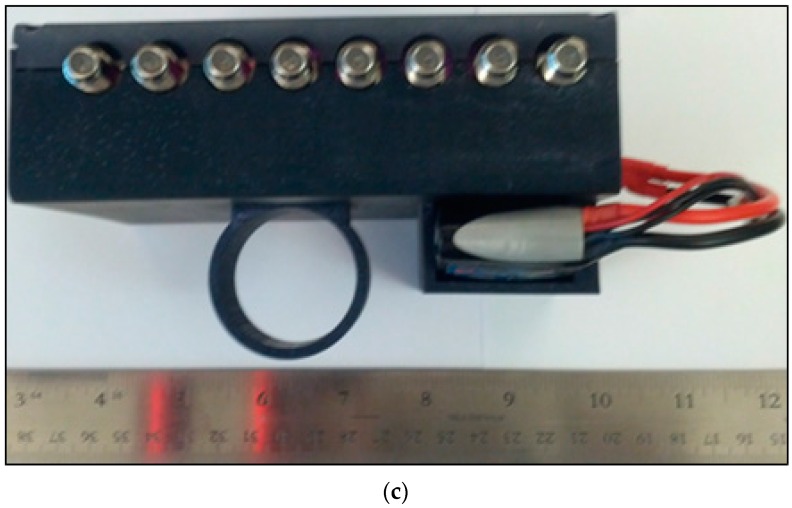
Acoustic goniometer enclosures; (**a**) Sensor enclosure; (**b**) Motherboard enclosure (Left) with sensor enclosure (Right); (**c**) Motherboard enclosure (side view).

In addition to needing a structure for supporting the sensors, the acoustic goniometer design further required enclosures to protect the sensors and motherboard from the elements. While not the ideal solution for outdoor environments, stereolithography (SLA) was used to print prototype cases for the acoustic goniometer. Figure 9a shows the first prototype enclosures, and Figure 9b,c show the second generation enclosures. The sensor board enclosure (Figure 9a) is slightly more complex than the motherboard enclosure (Figure 9c) as it has more of an impact on the system’s performance. Both enclosures include outlets for the coaxial connectors as well as cutouts for user interface accessibility (e.g., frequency selection slide switch on the sensor board and SD card cutouts on the motherboard). Each of the enclosures further includes mounting brackets designed to fit the PVC structure designed for the current prototype. However, the sensor board enclosure also includes a small wind screen to help combat noise generated by low speed winds (less than 10 mph). A small tube (not shown in the figure) extends both into the case to enclose the microphone and outside the case to provide a mounting point for the windscreen. Another key difference, other than size, (shown in Figure 9c) is the battery pocket on the back side of the motherboard enclosure. The only difference between the two generations of enclosures is the material selected. The second generation enclosures are still 3-D printed but are made from a more flexible material in order to prevent breakage caused by rough test procedures. The enclosures have been tested and proven reliable for a prototype but are not without drawbacks which would have to be addressed in a more permanent solution.

The SLA material suffers from several important drawbacks. First, cases printed in this fashion are expensive to obtain. Second, the material properties are not ideal and change over time and exposure to various environmental factors. The material when first printed is semi-rigid and breaks easily. Over time, the material continues to become more rigid and brittle. This process is accelerated when the material is exposed to any form of UV light. Consequently, this material is unsuited to outdoor applications in the presence of sunlight. What little flexibility the material has presents a problem to the goniometer in the form of increased wind sensitivity. The enclosures shown in the figure were tested on a fairly windy day (15 mph wind with gusts above 20 mph) with wind screens in place. Even with the wind screens, the enclosure’s material was just flexible enough to allow it to act like a speaker diaphragm in the presence of high speed wind (amplifying the infrasonic noise due to wind). The gun shots from this data can’t be separated from the wind noise since the frequency and amplitude of the signal generated by the wind is similar to those of the gun shot signals. The only positive feature of this type of enclosure is the quick turnaround of custom designs. This allowed multiple prototypes to be quickly developed for basic system tests. However, a more suitable material (e.g., more sturdy, weatherproof, windproof, *etc.*) should be selected for any long term deployments.

## 5. Field Tests and Results

### 5.1. Field Tests

**Figure 10:**
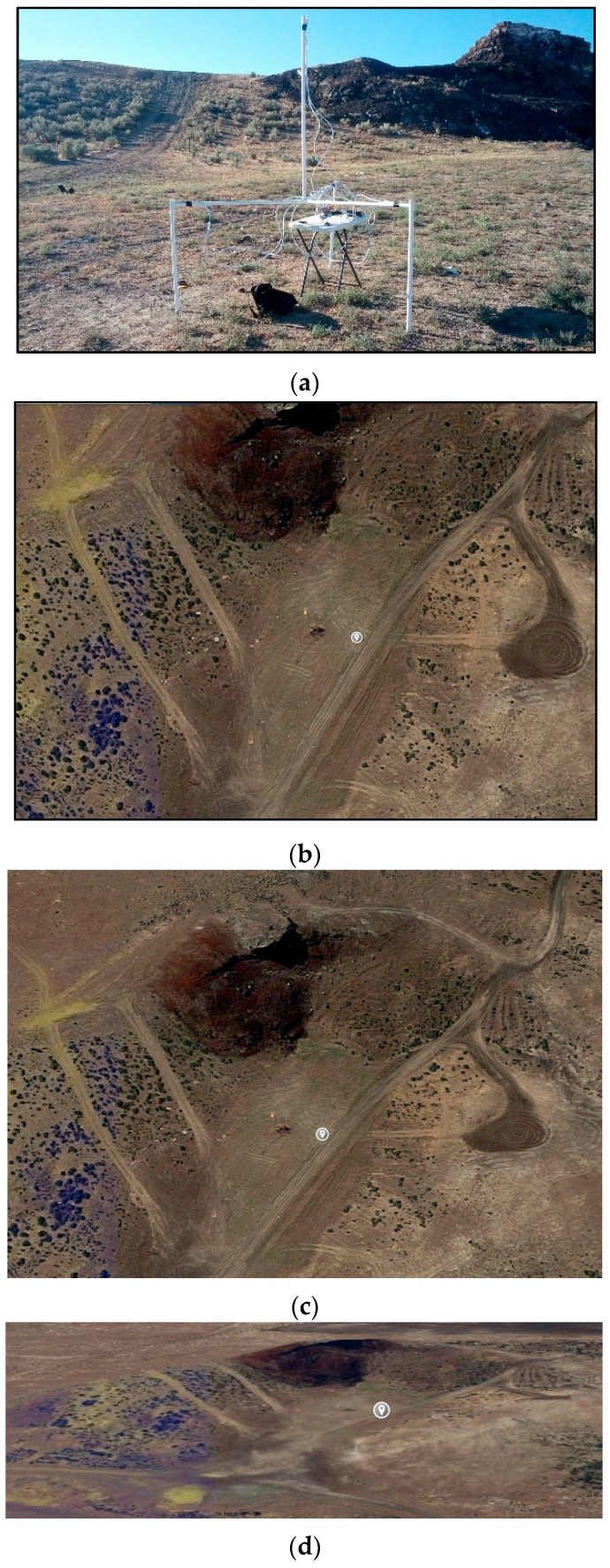
Goniometer field test terrain, (**a**) Acoustic goniometer in field; (**b**) Top-down satellite view of terrain (gray dot marks approximate goniometer location); (**c**) Slightly angled top-down satellite view of terrain (gray dot marks approximate goniometer location); and (**d**) Angled satellite view of terrain (gray dot marks approximate goniometer location).

The acoustic goniometer antenna was deployed in a dessert location surrounded by hilly terrain. Figure 10 shows the area surrounding the goniometer. The region is a small, elevated valley surrounded on every side except the left by hills (see Figure 10b). The hills provide a good backdrop for firing the AR-15 (hills supply a stopping point for bullets) and create a mild source of multipath error. While minimizing sources of error during testing is desirable, no natural environment is ideal. Thus, the multipath properties of this region were deemed acceptable for the goniometer tests. In addition to the multipath, another less than ideal feature of the site was the uneven ground upon which the tests took place. The images in Figure 10b–d make the valley inside the hills look fairly flat. However, close inspection of Figure 10a shows that the valley actually not only slopes down (toward the left side of the goniometer), but also that the area where the shots were fired has many small peaks and valleys of its own. While this did not affect the azimuth calculations in any appreciable manner, the uneven ground made determination of the elevation of the sound sources with the available equipment impossible. However, since the equations for azimuth and elevation are so closely related, verified functionality in the azimuth calculation guarantees similar performance in the elevation calculations.

**Figure 11:**
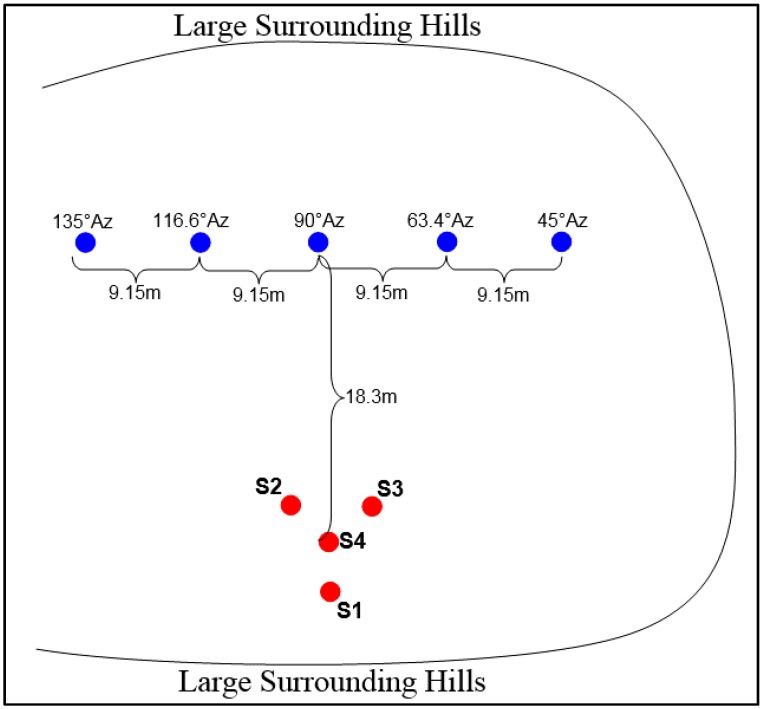
Field test event source layout.

Further, based on the small size of the peaks and valleys, an estimation of the elevation at each location to be within 2° of 0° was reasonable. Thus, this feature of the environment was also deemed acceptable. Once the goniometer was deployed, gun shots were fired by multiple researchers from various locations ranging from approximately 18 m to 26 m away from the center of the goniometer at angles from approximately 45° to 135° in azimuth at approximately 0° in elevation (±2° due to hilly terrain). The AR-15 was fired from a standing position by each researcher without the aid of a tripod. Figure 11 shows an approximation of the layout of the goniometer and event locations in the region shown in Figure 10. Since the use of survey equipment was not an option, in order to measure the angles, a simple grid was used to select locations from which to fire the AR-15. A point ~18.3 m from the center of the goniometer along a line running through the center and the leg of the antenna holding sensor 1 (S1: mic1) was selected as being the location for the 90° (azimuth) event source. Then, the remaining locations were selected at 9.14 m intervals along a line parallel to the one which can be drawn between sensor 2 (S2: mic2) and sensor 3 (S3: mic3). This method of measuring angles is not perfect but provided reasonable accuracy and proved adequate for the purpose of the test. However, in order to get more exact error measurements, these tests would have to be conducted with more precise measurements of the angles (both azimuth and elevation).

### 5.2. Results

Only a very small subset of the test results is shown in this paper, and extensive collected data can be found in [32]. The acoustic goniometer firmware performance during the field tests was good for all source angles tested. All of the calculations showed less than 5% error as compared to the expected approximate angles, and every event was properly identified and recorded with no false events. Results from the test data analysis is shown in Table 1. Since only azimuth was purposefully varied, elevation error is not shown in the table. The average error for the elevation of approximately 0° was 2.39%, and the worst error was 4.31%. The sources of error for this test include: Lack of survey equipment for measuring precise angles, approximated sensor locations on the prototype structure, finite numerical resolution. Given that antenna geometries have been shown to affect goniometer performance [13], the apparent favor shown by the system toward certain DOA angles is most likely due to the characteristics of the gunshot phenomena and the design of the goniometer’s antenna geometry. Based on the work done by Van Lanker [13], this explanation seems like the most valid theory, however further exploration into this behavior is worth considering. Overall, the acoustic goniometer performed well. The results show that the system is capable of being used for localization of events to within a reasonable tolerance. Further fine-tuning of the algorithm thresholds and windowing parameters could produce more accurate results.

## 6. Conclusions

The goal set forth for this research has been successful insofar as an inexpensive, small acoustic goniometer which is easy to deploy and capable of being adapted to meet the needs of a wide range of research applications while maintaining reasonable accuracy has been created. This work has effectively lowered several barriers to research by providing not only a working prototype but also documentation of the design process, tradeoffs, and guidelines for future expansion of the research and use of the acoustic goniometer.

The current firmware is flexible and provides efficient, accurate results. Furthermore, modification of the firmware is made simple by the current system in that the only requirement is the ability of the researcher to write/modify C code to fit their purpose. The algorithms selected to test the hardware are not the most advanced algorithms in the field, but they were adequate to the task of proving the system and achieved reasonable accuracy. Further, more advanced algorithms generally necessitate the research and characterization of a particular phenomenon in order to fully implement and evaluate their performance. Since this was outside the scope of the design of a general purpose goniometer which would allow more specialized research to be completed, such research was left for those interested in studying specific phenomena. The current firmware on the goniometer was successful for both DOA calculations and testing the capabilities of the hardware, and it allows room for and supports further exploration into more complex algorithms.

Design of a full implementation of the acoustic goniometer in firmware and embedded hardware has been completed, brought to fruition, and tested. The hardware chosen for this research was carefully selected after weighing all of the tradeoffs involved. While one could argue for the use of faster, more specialized processors, FPGA only implementations, or a combination of the two (system on a chip: SOC) for an improved design, the ease of continued development and modification had to be considered. More advanced hardware would provide resources to enable the use of more accurate algorithms and to increase sampling rates. However, these solutions would either greatly increase the cost of the system or create a more complex design requiring specialized skill sets to allow researchers to adapt the hardware for more specialized purposes. The current design allows for rapid reconfiguration to adapt the antenna (both spacing and geometry), frequency range, filters (both analog and digital), and sampling speed to meet the needs of a particular research project.

## Figures and Tables

**Table 1:** Goniometer performance analysis.

Azimuth	45°	63.4°	90°	116.6°	135°
**Avg. Error (%)**	1.64	1.24	1.48	2.25	1.9
**Worst Error (%)**	3.6	1.5	1.78	4.44	2.44
